# Adjunct Nondamaging Focal Laser Reduces Intravitreal Injection Burden in Diabetic Macular Edema

**DOI:** 10.3390/photonics10101165

**Published:** 2023-10-18

**Authors:** Lyna Azzouz, Asad Durrani, Yunshu Zhou, Yannis M. Paulus

**Affiliations:** 1 Department of Ophthalmology and Visual Sciences, University of Michigan, Ann Arbor, MI 48105, USA;; 2 Department of Ophthalmology, Stanford University, Palo Alto, CA 94305, USA; 3 Department of Biomedical Engineering, University of Michigan, Ann Arbor, MI 48105, USA

**Keywords:** diabetic macular edema, diabetic retinopathy, focal laser, Endpoint Management laser, anti-vascular endothelial growth factor

## Abstract

This study aims to determine the impact of adjunct nondamaging focal laser therapy on the number of anti-vascular endothelial growth factor (anti-VEGF) injections and visual acuity (VA) and imaging in patients with diabetic macular edema (DME). A retrospective analysis of 18 eyes of 14 patients with DME treated with a single session of the PASCAL 532 nm Synthesis Photocoagulator with Endpoint Management was conducted. Demographic data, VA, imaging, laser parameters, and anti-VEGF injection burden six months before and after treatment were collected. Wilcoxon Signed-rank tests were used to assess changes in VA and injection burden before and after treatment. The mean number of intravitreal injections in the six-month period prior to laser treatment was 3.39 ± 2.57 injections compared to 2.33 ± 2.40 injections following laser treatment (*p* = 0.02). There was no significant difference between the mean VA on the day of treatment logMAR VA of 0.38 ± 0.27 (approx. Snellen equivalent 20/50) and the visual acuity on the most recent follow-up 6 months after laser logMAR VA of 0.35 ± 0.32 (approx. Snellen equivalent 20/40) (*p* = 0.34). There was also no significant difference in OCT central macular thickness before (311 μm) compared to 6 months after (301 μm, *p* = 0.64). Adjunct focal macular laser therapy is associated with a statistically and clinically significant decrease in the number of intravitreal injections required in the six-month period immediately following treatment, without compromising visual acuity or macular thickness. Nondamaging focal laser has the potential to alleviate the burden of injections for both patients and clinics.

## 1. Introduction

Diabetic retinopathy (DR) is the leading cause of blindness in working-age adults in the USA, affecting approximately 4.1 million people, which is equivalent to 1 in 29 people [1,2]. In non-proliferative DR (NPDR), vaso-occlusion and increased vascular permeability can cause fluid extravasation, which may lead to diabetic macular edema (DME) [3,4], which is the most common cause of visual loss in patients with DR. Vascular endothelial growth factor (VEGF) plays an important role in this process.

Current therapies for DR include anti-VEGF intravitreal injections [5–9], laser treatments, intravitreal steroid injections, and vitreoretinal surgery. Laser treatments can be used to treat both PDR and DME [10,11]. Panretinal photocoagulation (PRP) has been shown to significantly reduce the risk of vision loss in patients with PDR, but it is also associated with numerous side effects, such as permanent retinal scars, patient discomfort, cystoid macular edema, and worse peripheral, color, and night vision [12]. The Early Treatment Diabetes Retinopathy Study (ETDRS) demonstrated that focal/grid laser treatment of the macula reduced rates of moderate vision loss in eyes with DME by 50% over a 3-year period [13].

Efforts to develop efficacious laser therapies for DME with a low side effect profile led to the creation of the semi-automated pattern scanning retinal photocoagulation system (PASCAL^®^, PAttern SCAn Laser; Optimedica Corp, Santa Clara, CA, USA) [14]. This system involves the rapid application of numerous spots (4 to 56 burns) in a defined pattern and with shorter pulse durations of 10–30 ms, which decreases the time of treatment and increases patient comfort and laser accuracy [12]. The Endpoint Management (EpM) laser is a newer non-damaging laser therapy designed to deliver the appropriate laser power to the macula even in the absence of visible tissue changes. The algorithm titrates laser power to cause changes in heat shock proteins (HSPs) without leading to permanent scarring and damage to the macula [15]. Stimulation of retinal pigment epithelial (RPE) cells is thought to increase the expression of heat shock proteins, which are chaperones for protein refolding, inhibit apoptosis, and decrease inflammation, overall improving the RPE function and reducing macular edema [16–18]. Studies have used transgenic mice expressing heat shock protein 70 (HSP-70) to determine retinal cell response to heat below the damage threshold. HSP-70 was expressed in the retinal pigment epithelium at energy levels of 25–30%, with little response at 20%. With a conventional laser, HSP-70 is detectable in the ring of cells surrounding the center of cell death, while with a subthreshold laser at 30% energy or less, HSP-70 was noted in the center of the laser spot, with no evidence of cell death [19,20].

Although studies have shown that intravitreal anti-VEGF injections result in better mean visual acuity (VA) than laser monotherapy [21,22], they require frequent [22], often monthly, treatment that poses a significant difficulty to patients [23,24] and their families and carries a rare but significant risk of endophthalmitis and other adverse effects [8,25]. Particularly given the COVID-19 pandemic in which many patients with diabetes are at high risk and have either deferred needed eye care or presented infrequently, this study sought to evaluate whether adjunct EpM may have the potential to reduce the number of injections and clinic visits needed for patients without compromising VA. Understanding the EpM laser’s effect on injection burden may aid in devising improved treatment plans using a more effective combination of EpM laser treatment and anti-VEGF injections.

## 2. Materials and Methods

Following IRB approval (HUM00180995), a retrospective review of all patients receiving EpM laser for the management of diabetic macular edema at the University of Michigan Kellogg Eye Center between June 2018 (when the laser was first acquired at our site) and June 2020 was conducted. Inclusion criteria included age 18 years or older, a diagnosis of DR and specifically DME, and management of the condition with a single session of EpM laser within the study period with a minimum follow-up time of 6 months. Exclusion criteria included eyes treated with EpM laser for non-DR pathologies, treatment with conventional focal laser photocoagulation, less than 6 months of follow-up, and inadequate clinical records.

Michigan Medicine patient records were queried with a diagnosis of diabetes mellitus and an American Academy of Professional Coders (AAPC) Current Procedural Terminology (CPT) code of focal laser treatment (67210) during the study period. Twenty potential focal laser patient records were reviewed, of which four were excluded due to the use of conventional focal laser. Sixteen patients were confirmed to have been treated with EpM laser over the target study period. Two patients were excluded due to inadequate records. However, one of the patients included in this study was new to the clinic with only three months of medical records at our institution and limited understanding of the previous therapies received. Given the limited sample of patients, we elected to extrapolate the patient’s number of intravitreal injections in a six-month period by doubling the number of injections received in the prior three-month period.

Data collected included demographic information, laterality of laser treatment, type of diabetes mellitus, type and severity of DR, time since diagnosis of diabetes mellitus, other ocular and systemic diagnoses, laser parameters, VA before and after laser treatment, central subfield macular thickness (CST) before and after laser treatment, number of injections before and after laser treatment, type of injection, and duration of follow up period. Snellen best corrected VA (BCVA) information from patient charts was converted to logMAR VA for statistical analysis. The severity of non-proliferative diabetic retinopathy was determined based upon the presence or absence of retinal bleeding, venous beading or other abnormal vascular findings, including (i.e., intraretinal microvascular anomalies or IRMA), using a combination of dilated fundus examination, Optos fundus photography, fluorescein angiography, and OCT of the macula. All patients were treated with one session of the PASCAL Synthesis Photocoagulator 532 nm with EpM (Iridex, Mountain View, CA, USA). Spectral domain Optical Coherence Tomography (OCT) was performed using the Zeiss Cirrus HD-OCT 5000 (Carl Zeiss Meditec, Inc., Dublin, CA, USA) cube centered on the fovea. The macula sparing the fovea and papillomacular bundle was treated with an EpM laser without specifically using OCT or FA images for targeting. The decision to treat with intravitreal injections and/or extend the interval between clinic visits was made on an as needed basis (PRN) for visually significant DME, was the same with all patients, and was the same before and after EpM laser, and included the following: 1. Visual acuity assessment and changes. 2. OCT thickness assessment and changes. 3. Patient and physician participation and decision-making on the role of the DME in affecting the patient’s vision.

### Statistical Analysis

The primary outcome of the study was the change in the number of anti-VEGF injections in the 6 months before and after EpM laser treatment. Secondary outcomes included changes in VA and in CST (measured on OCT) before and after laser treatment. All statistical analyses were conducted in SAS version 9.4 (SAS Institute, Cary, NC, USA). For qualitative variables, frequencies, and relative frequencies (in percentage) were calculated; for continuous variables, mean, standard deviation, and ranges were calculated. Wilcoxon Signed-rank tests were used to assess changes in numbers of injections, CST, and VA between post- and pre-EpM laser treatment. A *p*-value of < 0.05 was considered statistically significant.

Of note, calculating the percent change in injections, CST, and BCVA, before and after laser treatment, was conducted by first computing the percent change for each individual eye before obtaining a total mean. The alternative, computing total means before and after treatment first then using them to calculate a percent change, caused the eyes with larger baseline numbers (before laser treatment) to heavily skew the results.

## 3. Results

A total of 18 eyes from 14 patients were included in this study. The mean age at the time of treatment was 62.6 ± 17.1 years. Three patients were African American while the remaining eleven were Caucasian. Six of the fourteen patients were female. Twelve patients had type 2 diabetes, whereas two patients had type 1 diabetes. Eight patients had PDR, two had mild NPDR, two had moderate NPDR, and two had severe NPDR (Table 1). All patients were followed for a minimum of six months following the laser treatment. Based on a review of the electronic medical record and ophthalmology clinic notes, none of the patients had significant changes in systemic conditions over the course of the study period.

The EpM laser parameters are listed in Table 2. The mean number of intravitreal injections in the six-month period prior to laser treatment was 3.39 ± 2.57 injections compared to 2.33 ± 2.40 injections in the six months following laser treatment (*p* = 0.02). Twelve patients (50%) were receiving bevacizumab injections prior to laser treatment, seven (29%) patients were receiving aflibercept, and the remaining five were receiving a combination of anti-VEGF and steroid agents, with four receiving Ozurdex (dexamethasone intravitreal implant) and one patient receiving triamcinolone. Combination therapy with steroids and anti-VEGF was continued both before and after the EpM laser in all five patients. There is no statistically significant difference in CST measured on OCT on the last visit prior to treatment compared to 6 months after laser treatment (311.2 ± 78.0 μm compared to 301.4 ± 62.4 μm; *p* = 0.64). The mean VA on the day of treatment was logMAR VA of 0.38 ± 0.27 (Snellen equivalent 20/48) and at the most recent follow-up 6 months after laser treatment was logMAR VA of 0.35 ± 0.32 (Snellen equivalent 20/45), which was not statistically significant (*p* = 0.24) (Table 3).

### Demonstrative Case Example

The following case illustrates the clinical utility and impact of EpM laser in a real-world application: A 61-year-old Caucasian woman with DME and severe NPDR in both eyes presented to the University of Michigan Kellogg Eye Center retina clinic with new, constant blurry vision in the right eye. Her past ocular history was significant for glaucoma suspect, pseudophakia, and posterior vitreous detachments, all present in both eyes. Her past medical history was significant for diabetes mellitus type 2 for 20 years, sub-optimally controlled with a hemoglobin A1c of 10.2 (improved from 12), stage III chronic kidney disease, and hypothyroidism.

At presentation, BCVA in the right eye was 20/70 (pinhole to 20/40) compared to 20/25 in the left eye (Table 4). OCT demonstrated a CST of 347 μm. Her DME was worsening in the right eye and different treatment options were discussed.

At the time of this visit in the summer of 2020, the patient was very concerned about the risk of COVID-19 given her comorbidities. She was receiving monthly bevacizumab and the co-pay with aflibercept and ranibizumab was too expensive for her, so she wished to pursue other options. She was also concerned about using steroids given her glaucoma suspect status and her family history of advanced glaucoma. The EpM laser for this patient was cost-effective with a low side effect profile, so the patient elected to proceed with it to treat the right eye. The parameters used were the same as those in the study: 30% threshold with landmarks off, 200 μm spot size, 0.25 Φ space between spots, and pulse duration of 15 ms. The number of laser spots was 653, and the threshold power was 150 mW.

**Figure 1.**
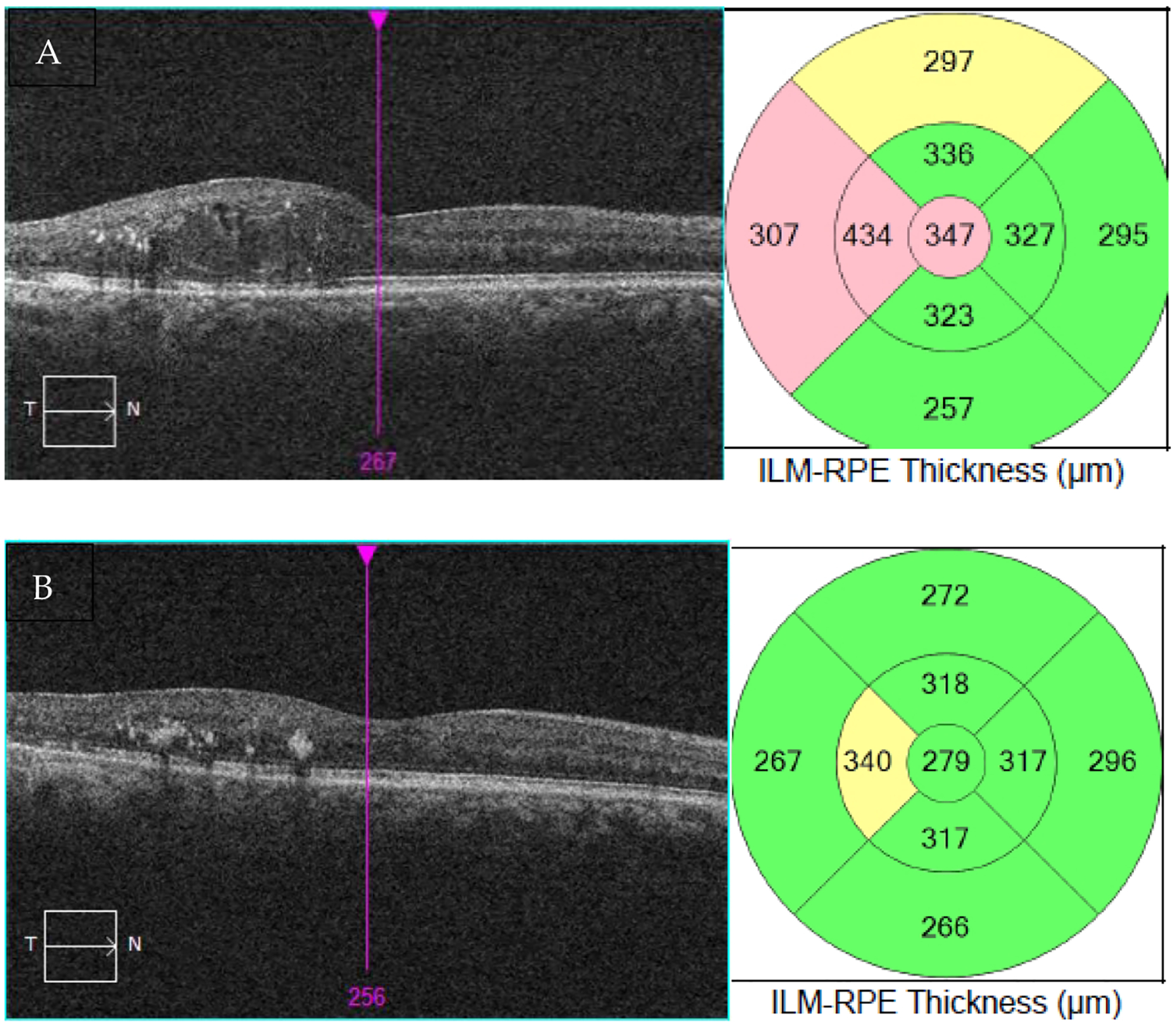
OCT of the macula and thickness measurement mapping at presentation (**A**) and after EpM laser therapy (**B**).

The patient was very pleased with the results, as she went from needing six intravitreal injections of bevacizumab in the 6 months before laser to two intravitreal injections in the 6 months after laser, with better vision and fewer frequent visits (Table 5). VA in the right eye improved from 20/70, ph 20/40 prior to EpM laser treatment to 20/30 after laser. The OCT of the macula also showed significant improvement (Figure 1), with a reduction in the thickness from 347 μm to 279 μm in the right eye. EpM laser treatment had resulted in improved VA while also reducing the injection burden and trips to the clinic, leading to a tangible improvement in the patient’s quality of life.

## 4. Discussion

This study aimed to determine the effect of adjunct nondamaging focal laser therapy using EpM on the number of intravitreal injections, BCVA, and CST in patients with DME. The results show that the EPM laser resulted in a significant decrease in the injection burden, without compromising visual acuity or macular thickness. The literature on the combination of sub-damaging laser therapies and intravitreal injections in DME largely focuses on subthreshold diode micropulse laser [26–33]. Micropulse laser and PASCAL with EpM laser are both considered subthreshold laser therapy. While the former involves short, repetitive pulses that last for microseconds, with significant cooling in between these pulses [34], the latter uses rapid administration of an array of laser spots (known as pattern scanning) with a shorter pulse duration, and the EpM software allows for precise control of power and duration and fine adjustment between visible and subvisible photocoagulation [19]. EpM offers precise titration that provides appropriate energy to stimulate RPE, short pulse duration and pattern scanning that is fast and reproducible, and the ability to produce barely visible burns that help with documentation of the treatment location and prevent unintended retreatment [19].

Several studies have found that the inclusion of micropulse lasers in the treatment of DME is associated with a significant decrease in intravitreal injection burden [32,33,35–38]. While many of those studies showed significant improvement in BCVA, some simply established the noninferiority of combination treatment with intravitreal injections and micropulse lasers compared to standard intravitreal injections-only therapy [29,36,38,39]. The association between micropulse laser and CST is less clear. Moisseiev et al. showed that subthreshold diode micropulse is also associated with a significant decrease in CST three months after intervention [40]; however, Kanar et al. did not show any significant changes in CST [29,35,39]. Inagaki et al.’s study did not show any significant differences between subthreshold micropulse laser and PASCAL laser with EpM [32], but overall the literature on the clinical use of EpM laser in patients with DME [41–43] is limited and most do not directly compare the number of injections before and after treatment.

Our study demonstrates a significant decrease in the number of intravitreal injections for DME following treatment with EpM laser and is the first to establish this association. The literature available on the topic, such as Hamada et al.’s study, reports improvements in retinal thickness but does not directly assess changes in the number of intravitreal injections after treatment. Our study did not find a significant change in macular thickness after laser treatment, although this is likely due to increases in CST leading to intravitreal injections in both groups with a PRN treatment strategy rather than fixed monthly injections. In addition, our patient sample includes CST < 300 μm, which translates into a floor effect with less dramatic change following laser treatment. Our results do corroborate the findings of other studies that the EpM laser does not compromise VA.

This study has some limitations. First, it is a retrospective study conducted at a single institution with a limited number of patients. Second, one of the patients included in this study was a new patient to the clinic with only three months of medical records available. Given the limited sample of patients, we elected to extrapolate the patient’s number of intravitreal as outlined in the methods section.

Further research is needed to better understand the role of the EpM laser in DM. Future directions may include investigating the effect of the EpM algorithm on subgroups of patients with DME, stratified by stage of DR so we can better assess the populations that would most benefit. The limited sample size in this study did not allow for such a subgroup analysis. Similarly, future research may explore the optimal frequency of repeat laser treatments with EpM as it may enable us to create a schedule involving both laser and anti-VEGF therapy at specific intervals or with the addition of steroid therapy.

## 5. Conclusions

The current gold standard for diabetic macular edema (DME) is frequent anti-VEGF injections [5–9]. The EpM laser has the potential to be a safe and effective method for reducing the injection burden in this group of patients while preserving visual acuity. Although our study did not find significant improvement in central macular thickness after laser treatment, a study with a larger number of patients or more restrictive macular thickness inclusion requirements may be needed to assess an association. Longer follow-up may be beneficial in assessing the durability of the laser effects. A reduction in the number of anti-VEGF injections offers tangible benefits as frequent intravitreal injections impart a considerable burden to patients [24,44], their families, and our healthcare system [45].

## Figures and Tables

**Table 1.** Patient characteristics.

Variable	No. (%) or Mean (SD)
Patients	
Total	14
Eyes	18
Age, years	62.6 (17.1)
Sex	
Male	8 (57.1)
Female	6 (42.9)
Race/ethnicity	
Caucasian	11 (78.6)
African American	3 (21.4)
Diabetes	
Type 1	2 (14.3)
Type 2	12 (85.7)
Eyes	
DR severity	2 (14.3)
Mild NPDR	2 (14.3)
Moderate NPDR	
Severe NPDR	2 (14.3)
PDR	8 (57.1)

Abbreviations: SD, standard deviation; DR, diabetic retinopathy; NPDR, nonproliferative diabetic retinopathy; PDR proliferative diabetic retinopathy.

**Table 2.** EpM laser parameters.

Percent of threshold	30% with landmarks off
Spot size	200 μm
Spacing of spots	0.25 Φ burn diameter apart
Pulse duration	15 millisecond
Mean number of spots	655.6 ± 160.5
Mean threshold power	147.3 ± 14.3 milliWatt

**Table 3.** Association of EpM laser treatment with changes in injection burden, macular thickness, and visual acuity.

Variable	Mean (SD)	Percent Change (%)	*p* Value
Number of injections			
Before EpM	3.39 (2.57)	−36 (0.0)	**0.02**
6 months after EpM	2.33 (2.40)		
Central subfield macular thickness, um			
Before EpM	311.2 (78.0)	0.4 (20.5)	0.64
6 months after EpM	301.4 (62.4)		
Visual acuity			
Before EpM	0.38 (0.27)	−14.0 (39.4)	0.24
6 months after EpM	0.35 (0.32)		

**Table 4.** Baseline eye exam findings of demonstrative case.

Exam Component	Right Eye	Left Eye
Best Corrected Visual Acuity	20/70, ph 20/40	20/25
Intraocular pressure (mm Hg)	12	14
Pupils	no RAPD	no RAPD

Abbreviations: ph, pinhole; RAPD, relative afferent pupillary defect; mm Hg, millimeter of mercury.

**Table 5.** Change in the number of injections in the 6 months before and 6 months after laser treatment, macular thickness on OCT, and visual acuity.

Variable	Pre-EpM Laser	6 Months Post-EpM Laser
Number of injections in 6 months	6	2
Central subfield macular thickness, μm	347	279
Best corrected visual acuity	20/70	20/30

## Data Availability

Data supporting reported results cannot be shared with the public due to privacy concerns.
